# Effect of Preoperative Oral Carbohydrate Intake on Perioperative Hyperglycemia in Indian Patients Undergoing Hip Fracture Fixation

**DOI:** 10.4274/TJAR.2024.231506

**Published:** 2024-05-03

**Authors:** Evelyn Eliza Minz, Rashmi Salhotra, Asha Tyagi, Aditya N. Aggarwal, Mohit Mehndiratta, S. V. Madhu, Venu George Toppo, Edelbert Anthonio Almeida

**Affiliations:** 1 Vardhman Mahavir Medical College & Safdarjung Hospital, Department of Anaesthesiology, New Delhi, India; 2 University College of Medical Sciences & GTB Hospital, Department of Anaesthesiology, New Delhi, India; 3 University College of Medical Sciences & GTB Hospital, Department of Orthopaedics, New Delhi, India; 4 University College of Medical Sciences & GTB Hospital, Department of Biochemistry, New Delhi, India; 5 University College of Medical Sciences & GTB Hospital, Department of Endocrinology, New Delhi, India; 6 Vardhman Mahavir Medical College & Safdarjung Hospital, Department of Community Medicine, New Delhi, India

**Keywords:** Blood glucose, carbohydrate loading, diet, enhanced postsurgical recovery, hip fractures, preoperative care/methods

## Abstract

**Objective:**

Preoperative fasting leads to a catabolic state aggravated by surgical stress. This leads to poor patient outcomes. This study aimed to determine the effect of preoperative oral carbohydrate administration on perioperative hyperglycemia and patient comfort.

**Methods:**

This prospective, randomized study was conducted on 60 adult American Society of Anesthesiologist I/II patients undergoing hip fracture fixation after obtaining institutional ethical committee clearance. Patients were randomly kept conventionally fasted before surgery (group F, n = 30) or were given oral carbohydrate 2 h before surgery (group C, n = 30). Under all aseptic precautions, a combined spinal epidural block was administered, and surgery was allowed. The primary outcome was blood glucose, and secondary outcomes included incidence of postoperative hyperglycemia, insulin level, blood urea, hunger, thirst, and anxiety.

**Results:**

Blood glucose levels were not statistically different between the two groups at baseline (T0; *P*=0.400), immediately after surgery (T1; *P*=0.399) and 24h after surgery (T2; *P*=0.619). The incidence of postoperative hyperglycemia was significantly higher in group F than in group C (*P*=0.045) at T2. Insulin levels, blood urea levels, and hunger scores were also not statistically different between the groups. The thirst and anxiety scores were lower at T0 and T1 in group C.

**Conclusion:**

Preoperative oral carbohydrate administration does not prevent perioperative increases in blood glucose levels. However, it reduces the incidence of perioperative hyperglycemia and decreases perioperative thirst and anxiety, thereby improving the quality of perioperative patient care.

**Main Points**

• Preoperative oral carbohydrate administration reduces the incidence of perioperative hyperglycemia.

• It improves patient comfort by decreasing thirst and anxiety.

• It enhances the quality of perioperative anaesthetic care.

## Introduction

Traumatic hip fractures are common in the geriatric demographics due to the prevalence of osteoporosis in this age group.^1^ These fractures typically require surgical intervention and implant insertion. A neuraxial blockade is the preferred method of anaesthesia, barring any contraindications. However, the perioperative period can be challenging because patients often suffer from comorbid conditions and malnutrition. These factors predispose them to an increased risk of surgical site infections, wound dehiscence, and prosthetic joint infections.

Conventionally, patients scheduled for surgery are required to undergo overnight preoperative fasting to prevent the risk of pulmonary aspiration during anaesthesia. Nevertheless, the triple insult of trauma, surgery, and prolonged preoperative fasting induces a catabolic state, leading to the production of several anti-insulin hormones such as catecholamine, cortisol, growth hormones, glucagon, etc.^2^ These hormonal changes contribute to the onset of stress hyperglycaemia^3^ and insulin resistance, which can last for up to 3 weeks.^4,5^ This state of hyperglycemia coupled with insulin resistance may be responsible for delayed wound healing, extended hospital stays, increased morbidity, and mortality.^6^ Furthermore, individuals of specific Southeast Asian ethnic origins have an increased susceptibility to postoperative hyperglycemia (POH).^7^ Thus, it is advisable to avoid prolonged periods of starvation in these patients.

The American Society of Anesthesiologists (ASA), the American Society for Parenteral and Enteral Nutrition, and the Enhanced Recovery after Surgery (ERAS) protocols recommend the administration of preoperative oral carbohydrate (POC)-containing fluids up to 2 h before surgery.

POC has become an integral part of the multidisciplinary approach within ERAS protocols for colorectal, cardiac, thoracic, and maxillofacial surgeries. However, the development of ERAS pathways for hip surgery is still ongoing.^8^ There is limited literature concerning the role of POC in patients of Southeast Asian descent undergoing hip surgery. However, existing evidence suggests that individuals of Indian ethnicity are more prone to develop POH than those of Chinese and Malay ethnicities. Therefore, this trial was designed to include Indian patients undergoing hip fracture fixation under combined spinal-epidural (CSE) anaesthesia. Although ERAS pathways have been recently introduced in more advanced medical facilities in the country, they are not yet widely adopted in smaller or peripheral setups. The primary objective of this study was to determine postoperative blood glucose levels, with secondary objectives including the incidence of POH, postoperative insulin levels, blood urea levels, patient comfort, and the quality of perioperative care in terms of hunger, thirst, and anxiety.

## Methods

This prospective randomized study was conducted at a tertiary care teaching facility in India from November 2019 to October 2021. Ethical approval for this study (IECHR/2019/41/3R) was granted by the Institutional Ethics Committee-Human Research on October 28, 2019, with Prof. Nalin Mehta serving as the chairperson. The trial was registered at ctri.nic.in (CTRI/2019/11/022084). All patients provided written informed consent before participation.

Sixty patients with ASA physical status I or II hip fractures, aged 18-65 years, were enrolled in the study. Patients with pre-existing conditions such as diabetes, pheochromocytoma, pancreatitis, alcoholism, jaundice or liver disorders, bleeding disorders, deranged coagulation profiles, local site infections, neurological or spinal diseases, obesity (body mass index >30 kg m^2-1^), gastroesophageal reflux, gastric outlet intestinal obstruction, pregnancy, or those who had undergone continuous steroid therapy for more than 5 days in the past year were excluded.

Patients were randomly allocated to two groups, Group F and Group C, using a computer-generated random number table. Randomization was performed using sequentially numbered sealed opaque envelopes. Patients in Group F (n = 30) followed the conventional fasting pattern and refrained from consuming any solids or liquids orally for at least 6 h before surgery. In contrast, patients in Group C (n = 30) abstained from consuming any solids orally for a minimum of 6 h but received 200 mL of Aptonia hydration powder (manufactured by Zeon Lifesciences Limited, Noida, Uttar Pradesh, India) 2 h before surgery. The weight of one sachet is 36 g. Each sachet of the powder contained a combination of sucrose, maltodextrin, dextrose, minerals, vitamins, acidity regulator (INS 330), anticaking agent (INS 551), added food color, and lemon flavor. The powder was dissolved in 300 mL of water, as recommended by the manufacturer, of which 200 mL (nearly 24 g) was administered to the patient.

Following a comprehensive pre-anaesthetic check-up, the patients were taken inside the operating room. Pulse oximetry, electrocardiogram, and automated noninvasive oscillometer blood pressure monitors were attached. An 18-gauge intravenous cannula was inserted, and Ringer’s lactate infusion was initiated. In addition, another cannula was inserted to collect blood samples.

With appropriate aseptic measures in place, CSE was administered in the midline lumbar region using 2.5-2.8 mL of heavy bupivacaine (0.5%). After ensuring an adequate level of subarachnoid block, surgery was started. Epidural top-ups with bupivacaine boluses were administered as necessary based on surgical requirements after a negative test dose. The remaining course of anaesthesia was performed according to the standard protocol.

Blood glucose, insulin, and urea levels were estimated at three time points: at the time of CSE administration (baseline; T0), immediately after surgery (T1), and 24 h postoperatively (T2). POH was considered when the blood glucose level exceeded 180 mg dL^-1^ during the postoperative period (T1 or T2). In addition, patients were enquired about their comfort levels related to hunger, thirst, and anxiety at both T0 and T1. Furthermore, hunger was evaluated using a subjective scoring system known as the “Hunger Level Scale,” while thirst was assessed using the “Perioperative Thirst Discomfort Score”.^9,10^ The presence of anxiety was noted as “yes” or “no”. Other parameters, such as admission-to-surgery interval, last-meal-to-surgery interval, last-liquid-to-surgery interval, intraoperative complications, length of hospital stay, 30-day readmission (defined as readmission to the hospital for related complications within 30 days of surgery), and 3-month mortality, were also documented.

### Sample Size and Statistical Analysis

Considering the immediate postoperative glucose levels as 105.40±17.47 mg dL^-1^ and 92.43±10.63 mg dL^-1^, and 24 h postoperative glucose levels as 115.31±23.78 mg dL^-1^ and 100.00±10.63 mg dL^-1^ in the no-POC and POC groups, respectively, as reported in a previous study,^11^ a sample size of 20 and 23 cases per group was required to estimate the same difference with a significance level (α) of 5% and a power of 80%. Because of the large sample sizes and to account for potential loss to follow-up, 30 cases were included in each group. Statistical calculations were performed using SPSS v.20.0 and STATA v.15.0. In addition, Student’s t-test, Mann-Whitney U test, repeated measures analysis of variance followed by Dunnett’s test, and chi-squared/Fisher’s test were employed as appropriate. Statistical significance was set at *P* value < 0.05.

## Results

**Figure 1:**
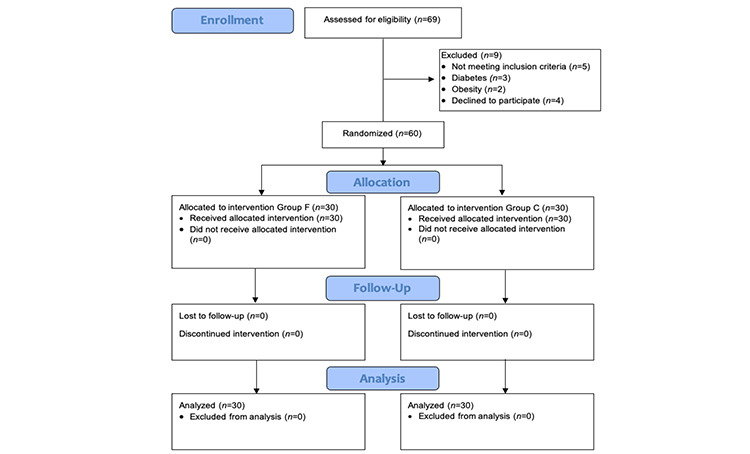
CONSORT flow diagram

A total of 69 patients were initially screened for eligibility. The CONSORT flow diagram of patient recruitment is shown in Figure 1. Among them, 60 patients were randomized, and all of them successfully completed the study protocol. The demographic characteristics of the patients included in the study were comparable between the two groups, as summarized in Table 1. Table 2 presents the surgical indications and fixation procedures performed.

A significant increase in blood glucose levels was observed in both the groups. However, blood glucose levels at corresponding time points were not statistically different between the two groups. Serum insulin levels at T2 were higher than those at T0 and T1 in Group F. Conversely, in Group C, insulin levels were higher at T2 than at T1 but remained not statistically different at T0 and T1 (*P*=0.472) and T0 and T2 (*P*=0.074). Insulin levels at the corresponding time points did not significantly differ between the two groups. Furthermore, no significant difference in urea levels was observed within or between the groups, as outlined in Table 3.

Table 4 lists the incidence of POH with blood glucose levels exceeding 180 mg dL^-1^ in both groups. None of the patients developed POH in the immediate postoperative period (T1). However, at the 24^th^ postoperative hour (T2), the incidence of hyperglycemia was notably lower in Group C than in Group F.

Table 5 provides the mean hunger and thirst scores, and the incidence of anxiety in the preoperative and postoperative periods. The mean thirst score was significantly lower in Group C than in Group F at both time points. Nonetheless, hunger scores in the preoperative and postoperative periods were similar between the two groups. Moreover, the incidence of anxiety was significantly lower in Group C than in Group F at both T0 and T1.

The interval between the last liquid intake and surgery was significantly shorter in Group C than in Group F (*P*=0.001). However, the time from admission to surgery (*P*=0.075), time from the ingestion of the last solid to surgery (*P*=0.811), and the length of hospital stay (*P*=0.348) were not statistically different among the groups. There was one incidence of bradycardia in Group C (3.33%) compared with none in Group F. Nine out of 30 patients in group F (30%) developed hypotension as compared to six out of 30 patients in Group C (20%). Also, the incidence of postoperative nausea and vomiting (PONV) was seen more in Group F (23.33%) as compared to Group C (3.33%). There was no incidence of 30-day readmission or 3-month mortality.

## Discussion

This study was conducted in 60 patients of Indian origin undergoing hip fracture fixation. The results revealed that POC administration did not mitigate the perioperative rise in blood glucose and insulin levels, which typically occur in response to surgical stress. However, it was effective in reducing the occurrence of POH in this population. Moreover, POC was effective in reducing the thirst experienced by patients during the perioperative period when adhering to conventional nil per oral guidelines. This approach also contributed to a reduction in anxiety levels among patients during the perioperative period.

Hip fracture is a common cause of hospitalization, surgical intervention, prolonged immobility, and increased morbidity and mortality among the elderly population. In our study, the mean age of the patients ranged from 36 to 40 years, reflecting the higher prevalence of hip fractures among younger age groups in our clinical setup. Notably, the crude incidence of hip fractures in India is substantial, with rates of 105 and 159 per 100,000 among men and women, respectively, accompanied by a one-year mortality of 42%.^12^ Given these alarming statistics, it is imperative to implement measures aimed at enhancing patient outcomes and reducing the burden on healthcare resources. Hip fractures include both intertrochanteric and femoral neck fractures, which are typically addressed through surgical interventions such as closed/open reduction with internal fixation using cortico-cancellous screws, dynamic hip screws, or proximal femoral nails. Nevertheless, prolonged preoperative fasting can predispose patients to a catabolic state as they struggle to tolerate the combined stress of trauma, surgery, and fasting.^4^ Consequently, patients may experience insulin resistance lasting up to 3 weeks.^5,6^

The POC administered in the study consisted of Aptonia hydration powder. This drink provided approximately 96 kcal of complex carbohydrates as opposed to those provided by canned fruit juices commonly used as preoperative drinks in previous studies.^13,14^ The composition aligned with the ideal drink recommended for preoperative administration. With a cost of only ₹20 ($0.24/€0.22) per sachet, it is affordable. Moreover, the drink is highly palatable and is available in lemon and orange flavors, with lemon-flavored powder being utilized in this study. POC was administered 2 h before the planned surgery to ensure gastric emptying before the administration of neuraxial block. In addition, the volume and timing of the drink conformed to the ASA and ERAS guidelines. A study has advocated the use of POC as it improves patient well-being and positively impacts glucose metabolism, insulin resistance, PONV, and pain management.^15^ In contrast, a meta-analysis of randomized controlled trials evaluating POC treatment in elective surgery observed that POC does not confer any beneficial effects on glucose clearance, insulin sensitivity, or postoperative complications.^16^ In addition, the latest ERAS guidelines for hip and knee surgeries do not categorize POC as an essential routine intervention, and its administration is left to the discretion of the anaesthesiologist.^17^

The Society for Ambulatory Anesthesia, American Diabetes Association, and Society of Critical Care Medicine recommend target blood glucose levels below 180 mg dL^-1^. Therefore, a cut-off value for hyperglycemia of >180 mg dL^-1^ was selected in this study.^18,19,20^ Generally, blood glucose levels increase during the perioperative period owing to surgical stress.^21^ The findings revealed that POC was not very effective in preventing this increase. However, spikes exceeding 180 mg dL^-1^ were less frequent among patients who received POC. Thus, POC effectively prevents the risk of blood glucose reaching harmful levels, which are associated with adverse perioperative outcomes.

Insulin levels similarly increased in the postoperative period, but values in the POC group consistently remained lower than those in the fasting group, suggesting the beneficial effect of POC. Because POC administration largely prevents the catabolic process to a great extent,^22,23^ insulin levels in the blood are thereby reduced, leading to a decrease in insulin resistance. Furthermore, a systematic review examining the role of POC observed its ability to mitigate insulin resistance.^24^ However, a sample size of 33 patients per group is required to obtain a statistically significant difference in insulin levels at 80% power and α = 5%.

In addition, increased blood urea levels in the postoperative period can serve as an indicator of nitrogen breakdown,^25^ poor renal function, or acute kidney injury.^26^ Insulin stimulation may also increase urea clearance.^27^ Given that postoperative insulin levels were higher than preoperative levels in our study, it is plausible that insulin-stimulated urea clearance may have increased. Therefore, postoperative blood urea levels may have remained within a range comparable to preoperative values.

Preoperative hunger, thirst, and anxiety are common measures of patient comfort, satisfaction, and overall quality of care during the perioperative period.^28^ Thirst can lead to feelings of anxiety, irritability, and weakness.^28^ Moreover, discomfort due to thirst may be intensified in tropical countries such as India, where temperatures are often high. Hence, implementing a liberal protocol of fluid administration up to 2 h before surgery may improve patient comfort and decrease anxiety levels. However, it should be noted that the results of the reduction of anxiety and thirst with POC administration are not universal.^15^ In our study, we observed a lower incidence of preoperative thirst as well as preoperative and postoperative anxiety in the POC group than in the fasting group; however, hunger scores were not statistically different between the two groups. These findings align with those of previous studies reporting inconsistent results regarding hunger levels following the administration of carbohydrate drinks. For instance, a study performed on patients undergoing gynecological laparoscopic surgery did not observe reduced hunger in those receiving POC,^29^ which agrees with our findings. In contrast, according to Imbelloni et al.^28^, patients who received POC were not hungry as they received the drink twice: once 2-4 h before surgery and again after spinal block inside the operating theatre.^28^ Our results on hunger levels can be attributed to the fact that the Aptonia solution provided only 96 kcal of energy, and hunger assessments were conducted 2 h after consuming this drink. It is possible that the calories provided by the drink were not sufficient to satisfy the hunger of an adult patient who had fasted overnight.

This study determined the efficacy of POC on glucose and insulin levels during the perioperative period. To adhere to the ERAS pathway, we opted against the use of general anaesthesia and opioids during the perioperative period. Instead, we employed epidural catheters for perioperative and postoperative analgesia, specifically for prolonged surgeries, to ensure adequate pain relief and maintain patient comfort. Meanwhile, PONV was actively monitored and promptly addressed with appropriate treatment whenever it occurred. In addition, perioperative patient comfort was measured in terms of objective hunger and thirst scores, providing a more comprehensive evaluation than previous studies that only assessed the presence or absence of these symptoms.

### Study Limitations

In addition, the study has certain limitations that should be acknowledged. Despite observing a lower incidence of PONV in patients receiving POC,^30^ the study was not adequately powered to detect differences in PONV. Therefore, any potential differences in PONV between the groups may not have been readily clear. Additionally, while the ERAS protocol is known to reduce the length of hospital stay, only a portion of this protocol was implemented in our study through the administration of POC. Therefore, no beneficial effects on the length of hospital stay were observed.^31,32^ Furthermore, insulin resistance could not be accurately calculated in our study because of the limitations in our setup. Specifically, the hyperinsulinaemic clamp technique, which is considered the gold standard for dynamic insulin resistance assessment, was not feasible to implement. Moreover, the homeostatic model assessment of the insulin resistance equation could not be applied because it measures static insulin resistance and may not accurately reflect the situation in the perioperative setting. The potential clinical benefits of POC about surgical site infections, renal failure, and cardiovascular events were also not evaluated.

## Conclusion

Based on the findings of this study, it can be concluded that POC administration does not effectively prevent the perioperative increase in blood glucose levels, although it leads to a modest increase in insulin levels among Indian patients undergoing hip surgery under neuraxial block. Despite this, the practice of administering POC remains beneficial as it reduces the incidence of POH and improves patient comfort by considerably alleviating perioperative thirst and anxiety. Therefore, we recommend the administration of 200 mL POC containing maltodextrin and a suitable flavoring agent up to 2 h before surgery, particularly in countries like India where the climatic conditions are hot and thirst levels are prevalent. This intervention can improve the overall quality of perioperative care.

## Figures and Tables

**Table 1:**
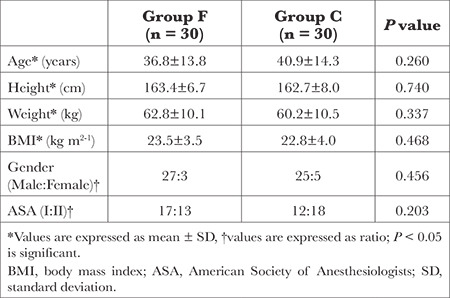
Demographic Characteristics of the Patients

**Table 2:**
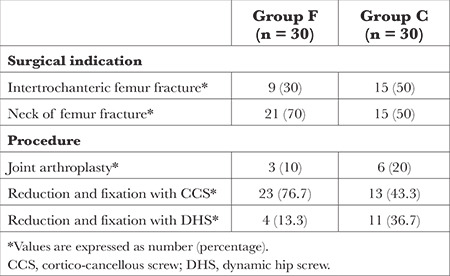
Surgical Indications and Surgical Procedures

**Table 3:**
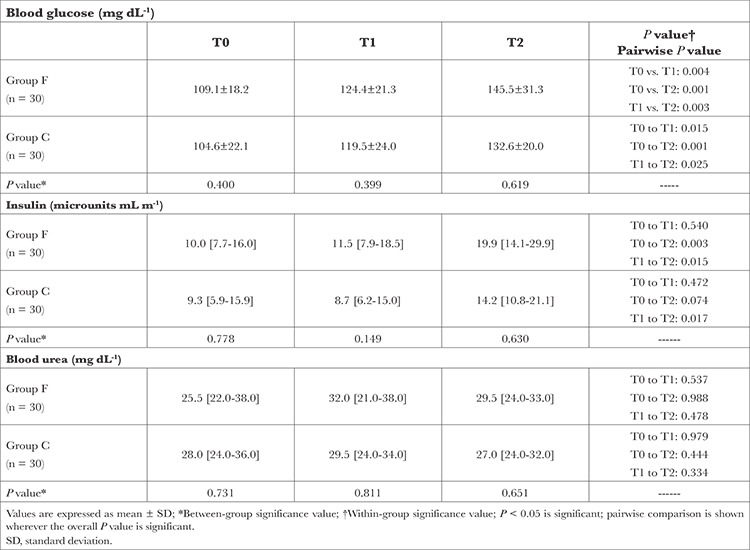
Comparison of Biochemical Markers in the Perioperative Period

**Table 4:**
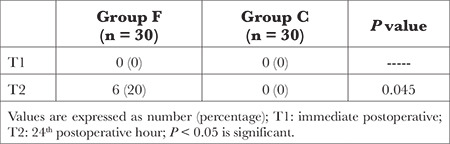
Incidence of Postoperative Hyperglycaemia

**Table 5:**
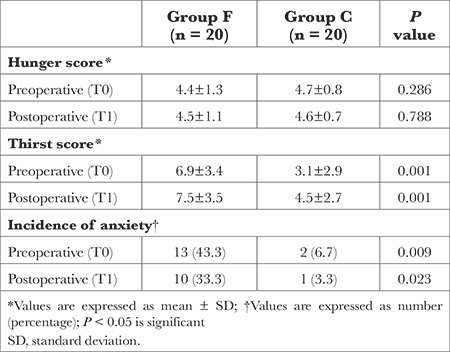
Hunger Score, Thirst Score, and Incidence of Anxiety
